# An Overview of the Molecular Genetics of Plant Resistance to the Verticillium Wilt Pathogen *Verticillium dahliae*

**DOI:** 10.3390/ijms21031120

**Published:** 2020-02-07

**Authors:** Ranran Song, Junpeng Li, Chenjian Xie, Wei Jian, Xingyong Yang

**Affiliations:** Chongqing Engineering Research Center of Specialty Crop Resources and The College of Life Science, Chongqing Normal University, Chongqing 401331, China; s13628213851@163.com (R.S.); ljpW0501@163.com (J.L.); jianwei7956@163.com (W.J.)

**Keywords:** plant resistance, resistance-related genes, vascular plant diseases, *Verticillium dahliae*

## Abstract

*Verticillium dahliae* is a soil-borne hemibiotrophic fungus that can lead to plant vascular disease and significant economic loss worldwide. Its hosts include over 400 dicotyledon plant species, such as annual herbs, perennials, and woody plants. The average yield loss of cotton crop caused by *Verticillium* wilt is approximately 10–35%. As the control of this disease is an urgent task for many countries, further understanding of the interaction between plants and *V. dahliae* is essential. Fungi can promote or inhibit plant growth, which is important; however, the most important relationship between plants and fungi is the host–pathogen relationship. Plants can become resistant to *V. dahliae* through diverse mechanisms such as cell wall modifications, extracellular enzymes, pattern recognition receptors, transcription factors, and salicylic acid (SA)/jasmonic acid (JA)/ethylene (ET)-related signal transduction pathways. Over the last decade, several studies on the physiological and molecular mechanisms of plant resistance to *V. dahliae* have been undertaken. In this review, many resistance-related genes are summarised to provide a theoretical basis for better understanding of the molecular genetic mechanisms of plant resistance to *V. dahliae*. Moreover, it is intended to serve as a resource for research focused on the development of genetic resistance mechanisms to combat *Verticillium* wilt.

## 1. Introduction

The soil-borne hemibiotrophic phytopathogenic fungus *Verticillium dahliae* can cause refractory vascular Verticillium wilt in a wide range of crops worldwide due to its highly aggressive pathogenicity and production of melanised dormant structures called microsclerotia, which can survive for several years in the soil [1]. It produces cell wall-degrading enzymes and phytotoxins, which cause signs of the disease. The fungus infects more than 200 dicotyledon plant species, such as annual herbs, perennials, and woody plants. The average yield loss of cotton crop caused by Verticillium wilt is approximately 10–35% in many countries. It generally causes plant dysplasia, leaf wilt, and yellowing and browning of vascular bundles, eventually leading to early death in some plants. At present, there are no fungicides available to control the infected plants [2], and thus Verticillium wilt results in extensive economic losses [3].

*V. dahliae* can infect a variety of dicotyledonous species including cotton, tobacco, tomatoes, *Arabidopsis*, and others. It usually invades and colonises the roots of plants, and then spreads throughout the plant [4]. *V. dahliae* begins to infect the roots of the plant through the soil, and hyphae penetrate the surface of the plant roots to colonise the vascular bundles, leading to plant death [5,6].

The main mechanism of its pathogenesis is xylem vessel blockage and toxin production. When the fungus invades the plant body, the mycelium blocks the xylem vessel, affecting the transport of water and nutrients in the plant [1,7]. However, transpiration and respiration of the aerial part are strong, causing water imbalance in the plant and signs such as leaf wilting and yellowing, which eventually leads to plant death [8]. However, catheter blockage is not the primary cause of plant wilting [7,9]. In the toxin theory, histological evidence indicates that leaf necrosis is caused by the action of mycotoxins [10]. The toxin produced by *V. dahliae* is an acidic protein–lipopolysaccharide complex [11]. It can seriously damage the metabolism of the plant body, fix carbon dioxide, decompose H_3_PO_4_, and eventually lead to plant death [12]. Current research indicates that toxin production is the main cause of plant wilting [2,13].

**Figure 1:**
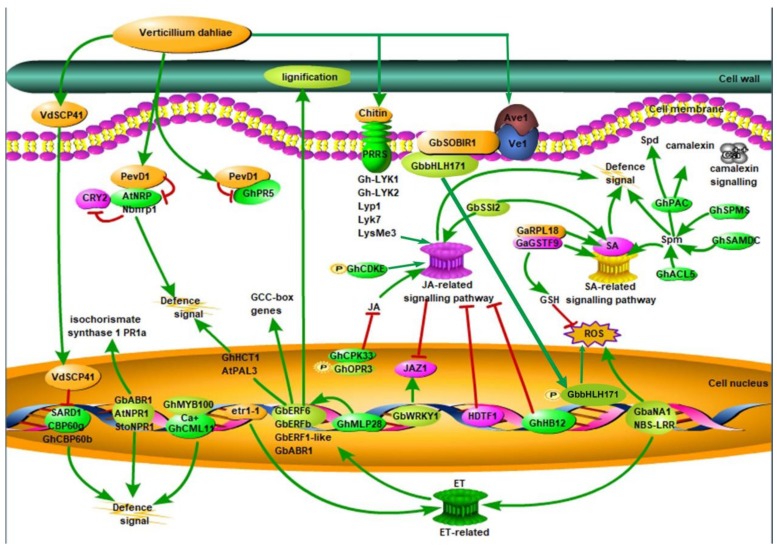
Regulation of intracellular signalling-related genes and signal transduction-related genes in response to *Verticillium dahliae* in plants. Plants have developed a sophisticated immune system to defend against *V. dahliae*. Plant cells immediately trigger signal transduction, leading to a rapid defence response including large-scale transcription reprogramming, while they recognise microbial-related molecular patterns or internal effectors from *V. dahliae*. 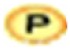, phosphorylation; CRY2, cryptochrome 2; ET, ethylene; GCC-box, ethylene-responsive element binding factor associated amphiphilic repression domain; GSH, phi-class glutathione; HDTF1: homeodomain transcription factor gene 1; JA, jasmonic acid; JAZ1, Jasmonate Zim-domain1; NBS-LRR, nucleotide-binding site leucine-rich repeat; PevD1, an elicitor from *V. dahliae*; SARD1, the *Arabidopsis* master immune regulator; SA: salicylic acid; Spd, spermidine; Spm, spermine; ROS, reactive oxygen species; SA, salicylic acid; VdSCP41, a secretory protein from *V. dahliae* [7,16,17,18,19,20,21,22]. Red lines represent negative regulation and green lines represent positive regulation.

Plants have evolved numerous defence mechanisms to protect themselves from invading pathogens [14], and plant extracellular enzymes and the cell wall are the first defence barriers. Subsequently, plants induce pathogen-associated molecular pattern (PAMP)-triggered immunity by recognizing pathogens using cell-surface pattern recognition receptors (PRRs). In turn, pathogens have evolved mechanisms such as effectors to overcome these PAMP-induced defence mechanisms. The effector is recognised by plants to activate effector-triggered immunity (Figure 1). The plant immune defences have been described as a ‘zigzag’ model, in which many genes are involved in this ‘zigzag’ procedure [15].

Functional analysis of the key genes involved in growth and pathogenicity is the molecular genetic basis of revealing plant resistance to *V. dahliae*. Currently, several studies focusing on plant resistance to *V. dahliae* have been reported. In this review, some key findings and resistance-related genes are summarised to provide a theoretical basis to further understand the molecular genetic mechanisms of plant resistance to *V. dahliae*.

## 2. Defence-Related Proteins

In plants, defence-related proteins play a significant role in plant resistance to fungal pathogens. Polygalacturonase, which digests pectin in plant cell walls, contributes to fungal pathogenicity and plays a considerable role in the pathogenicity of *V. dahliae* [23]. A class of plant defence proteins, polygalacturonase-inhibiting proteins (PGIPs), can specifically inhibit endo-polygalacturonases. Further, the overexpression of *CkPGIP1* from *Cynanchum komarovii* and *GhPGIP1* from *Gossypium hirsutum* in cotton can improve cotton resistance to *V. dahliae*, which is associated with the upregulated expression of pathogenesis-related proteins (PRs), enhanced disease susceptibility 1 (EDS1), phytoalexin-deficient 4 (PAD4), and isochorismate synthase 1 (ICS1) genes that enhance xylem lignification [24,25].

NaD1, a plant defensin from *Nicotiana alata* with strong antifungal activity against many filamentous fungi, is associated with significant resistance to *V. dahliae* after transgenic expression in cotton plants [26]. *GbNRX1* gene codes an apoplastic thioredoxin protein from *Verticillium* wilt-resistant island cotton (*G. hirsutum cv* Hai 7124), which is associated with an increase in abundance in response to *V. dahliae* infection. The observed increase in apoplastic reactive oxygen species (ROS) accumulation and reduced *V. dahliae* resistance in *GbNRX1*-silenced plants suggest that *GbNRX1* can scavenge apoplastic ROS and is pivotal for the apoplastic immune response [27]. Hydroxyproline-rich proteins (HyPRPs) comprise a plant cell wall glycoprotein subfamily enriched in proline. The GbHyPRP1 protein from *Gossypium barbadense* contains proline-rich repetitive and Pollen Ole e I domains and negatively regulates the resistance of cotton plants to *V. dahliae*. *GbHyPRP1* silencing was shown to markedly enhance cotton plant resistance to *V. dahliae* via cell wall thickening and ROS accumulation [28].

Non-expressor of pathogenesis-related protein 1 (NPR1) is a key regulator of systemic acquired resistance (SAR) in plants. When plants lack functional NPR1, their ability to express the PR gene is impaired and they show a near-total lack of an SAR response to pathogen infection [29]. Constitutive expression of the *Arabidopsis* NPR1 (*AtNPR1*) gene in cotton significantly increases the resistance of transgenic plants to non-defoliating *V. dahliae* [30]. *StoNPR1*, a *Solanum torvum NPR1* gene, was previously expressed in *V. dahliae*-sensitive potato, which increased the resistance of transgenic plants to *V. dahliae*. Further, *ICS1* and *PR1a* expression was also evidently enhanced in the *StoNPR1* overexpression lines and was significantly induced by *V. dahliae* infection [31]. GhMLP28, a defence-related major latex protein (MLP) from *G. hirsutum*, is induced by *V. dahliae*, jasmonic acid (JA), salicylic acid (SA), and ethylene (ET). *GhMLP28* silencing enhances the susceptibility of cotton plants to *V. dahliae* infection, whereas *GhMLP28* ectopic overexpression in tobacco increases disease resistance. A further assay demonstrated that GhMLP28 activates the transcription factor activity of ET response factor 6 (GhERF6), which augmented the expression of some GCC-box (AGCCGCC element) genes, contributing to defence against *V. dahliae* [32].

Sulphur plays a considerable role in tomato disease resistance against *V. dahliae*. The expression of genes related to sulphur uptake and assimilation, sulphur-containing defence compounds, and high-affinity sulphate transporter genes are increased in *V. dahliae*-infected tomatoes during companion cropping [33]. The haumatin-like protein (*TLP*) gene is related to plant biotic and abiotic stress regulation. Transgenic plants with higher expression of the cotton TLP gene (*GbTLP1)* show enhanced resistance to different stress factors including *V. dahliae* infection [34]. A synthetic non-cyclic 0200-defensin derivative, BTD-S, shows robust antimicrobial activity to *V. dahliae* in vitro [35]. Further, the expression of BTD-S in *Arabidopsis thaliana* increases resistance to *V. dahliae* [36].

*StoCYP77A2* is a wild eggplant cytochrome P450 gene and is induced by *V. dahliae*. Constitutive expression of *StoCYP77A2* in tobacco enhances plant resistance to *V. dahliae* infection. Protein extraction from *StoCYP77A2*-transgenic tobacco indicated strong antifungal activity, which implies that *StoCYP77A2* should participate in the synthesis of some antifungal compounds [37]. *GhDIR1* encodes a putative dirigent protein and its overexpression leads to increases in lignin content in transgenic cotton plants, which display enhanced tolerance to *V. dahliae* infection [38]. *GhUMC1*, an umecyanin-like gene in cotton, is involved in the resistance of cotton plants to *V. dahliae* through regulation of the JA signalling pathway and lignin metabolism [39].

## 3. Enzymes

In plants, extracellular enzymes are often the first line of defence against fungal pathogens. Increasing evidence shows that chitinase is a key hydrolytic enzyme, which degrades the fungal cell wall [40], and its expression can be initiated in response to biotic and abiotic stress [41]. Chi28 belongs to the class IV chitinase subfamily, and *Chi28* silencing significantly impairs cotton plant resistance to *V. dahliae.* VdSSEP1, a secretory serine protease, was shown to hydrolyse Chi28; however, cotton apoplastic protein CRR1 protects Chi28 from VdSSEP1-induced cleavage [42].

Pectins as the main element of the primary plant cell wall play a key role in defence mechanisms against plant pathogens. Pectin methylesterases (PMEs) catalyse dimethyl esterification of the homogalacturonan domains of pectin in the plant cell wall. *GhPMEI3* silencing in cotton leads to enhanced susceptibility to *V. dahliae*. Moreover, GhPMEI3 and GhPMEs might participate in protein–protein interactions and are important for plant evolution to resist fungal pathogens [43].

Lignification in the plant cell wall is a plant innate immune defence response and the lignification of lignin in resistant cotton stems contributes to the resistance of cotton to disease [44]. *GhLAC15*, a laccase gene, was demonstrated to be strongly induced by pathogens. Moreover, its overexpression increases Verticillium wilt resistance via increased defence-induced lignification and arabinose and xylose accumulation in the upland cotton cell wall [45].

The expression of *GbSBT1* in *G. babardense,* which encodes a subtilase that is mainly localised to the cell membrane, is induced by *V*. *dahliae*, JA, and ET, as it translocates to the cytoplasm following JA and ET treatment. *GbSBT1* gene silencing reduces the tolerance of Pima-90 (resistant genotype) to *V*. *dahliae* infection. Moreover, the overexpression of *GbSBT1* activates the expression of defence-related genes and increases *Arabidopsis* resistance to *Fusarium oxysporum* and *V*. *dahliae* [46].

Enoyl-CoA reductase (ECR) plays a crucial role in very-long-chain fatty acid formation. *GhECR*-silenced cotton plants are susceptible to *V. dahliae* infection, indicating that the *GhECR* gene is related to cotton resistance to different *V. dahliae* strains [47]. Cotton GbANS contributes to anthocyanin biosynthesis, and *GbANS* silencing significantly reduces anthocyanin production and cotton plant resistance to *V. dahliae* [48]. A U-box E3 ubiquitin ligase, GhPUB17, which can interact with and is inhibited by the antifungal protein GhCyP3, negatively regulates cotton resistance to Verticillium wilt pathogen [49]. The production of gossypol is sufficient to influence the resistance of cotton plants to *V. dahliae*. As such, silencing *GbCAD1*, encoding a key enzyme involved in gossypol biosynthesis, compromises cotton plant resistance to *V. dahliae* [50].

## 4. Receptor-Like Proteins

Lysin motif (LysM)-containing proteins are important PRRs in plants, which function in chitin recognition and the activation of defence responses against fungal pathogen attacks [51,52,53]. GhLYK1 and GhLYK2, two LysM-containing proteins, are induced after *V. dahliae* infection. However, *GhLYK1* and *GhLYK2* silencing compromises cotton plant resistance to *V. dahliae*. *GhLYK2*, but not *GhLYK1*, can induce ROS bursts in plants. Therefore, *GhLYK2* and *GhLYK1* might be distinctively dedicated to cotton defence [54]. In addition, in cotton, three important PRRs (Lyp1, Lyk7, and LysMe3) play an important role in activating downstream defence processes and inducing the defence response to *V. dahliae* via the recognition of chitin signals. The three PRR proteins are induced in response to *V. dahliae*, and their silencing greatly impairs SA, JA, and ROS generation, as well as resistance to *V. dahliae* [53].

In tomato (*Solanum lycopersicum*), *Ve* encodes receptor-like proteins containing extracellular leucine-rich repeats, and the *Ve* R-gene locus contributes to *Verticillium* resistance [25]. *Ve1* is involved in the race-specific resistance to *Verticillium* wilt pathogen infection [55]. The *Ve* locus includes two closely-linked inverted genes, *Ve1* and *Ve2*, encoding the extracellular leucine-rich repeat receptor-like protein (eLRR-RLP) and cell surface receptors [16]. Furthermore, it was shown that *Ve* genes encode a class of cell-surface glycoproteins with leucine zipper and receptor-mediated endocytosis-like signals [56]. Antagonistic relationships exist between Ve1 and Ve2 proteins, in which Ve1 modulates the induction of defence/stress proteins by Ve2 [25]. However, *Ve1* transgene introduction does not alter the endogenous *Ve2* expression [57].

Ve1 mediates plant resistance by monitoring the presence of the Ave1 effector in *V. dahliae* [58]. Some results suggested that H_2_O_2_, peroxidase, lignins, phenylalanine-ammonia lyase (*PAL*) gene expression, and JA are required for *Ve1*-mediated resistance to *V. dahliae* [59,60]. *Ve1*-transgenic *Arabidopsis* is only resistant to race 1, but not to race 2, strains of *V. dahliae*, *V. albo-atrum*, and *V. longisporum*. Importantly, the critical elements for resistance signalling are conserved, and the signalling components ACIF, MEK2, SERK3/BAK1, and SERK1 play a role in Ve1-positive regulation [61]. The defence signalling cascade downstream of Ve1 is required by ACIF, EDS1, NRC1, NDR1, MEK2, and SERK3/BAK1. *Ve1*-mediated plant defence requires the basal defence signalling elements *EDS1,* NRC1, and *NDR1* [61,62]. The constitutive expression of tomato Ve1 in *Arabidopsis*, cotton, and tobacco plants results in increased resistance to *Verticillium* wilt [63]. As a consequence of Ve1/Ave1-induced immune signalling, the immune receptor Ve1 recognises the *V. dahliae* effector protein Ave1 and then triggers a hypersensitive response in tobacco and tomato [64], but it is not entirely required for *Verticillium* resistance [65].

Phylogenetic analysis also indicates that Ve1 homologues are extensively scattered in land plants, and that Ve1 homologues in hop (*Humulus lupulus*), potato (*S. tuberosum*), tobacco (*Nicotiana glutinosa*), and wild eggplant (*S. torvum*) have been cloned and characterised [66]. *Gbve1*, a tomato *Ve* homolog, was cloned from an island cotton cultivar with resistance to *Verticillium* wilt and it can be induced by *V. dahliae* infection via SA, JA, and ET. *Gbve1* silencing in resistant cotton decreases the resistance to *V. dahliae*. Conversely, the overexpression of *Gbve1* in *Arabidopsis* and upland cotton plants enhances resistance to *V. dahliae* [67].

*Ve* homologous genes, *Gbvdr3 and Gbvdr6,* encode plasma membrane receptor-like protein in the *Verticillium* wilt-resistant cotton cultivar *G. barbadense* Hai7124 [68,69]. Silencing and overexpression experiments suggested their involvement in cotton resistance against *V. dahliae* and that they can enhance transgenic cotton or *Arabidopsis* resistance to *V. dahliae*. Their expression is activated by SA, methyl jasmonate, abscisic acid, and ET, and is induced by *V. dahliae*. In transgenic *Arabidopsis*, the overexpression of *Gbvdr3* and *Gbvdr6* enhances the expression of JA/ET signalling pathway-related genes ethylene-responsive factor 1 (*ERF1*), *PR3*, and *PDF 1.2*; SA-related genes *PR1* and *PR2*; the ET-regulated gene *GST2*; and ROS and callose accumulation in the early stage after *V. dahliae* infection [68,69,70].

A *Ve1* homologous gene, *VvVe,* was identified in *Vitis vinifera*, and its overexpression in tobacco significantly increased resistance to *V. dahliae* and upregulated defence-related gene expression, including the SA-regulated pathogenesis-related protein gene (*PR1*), ET- and JA-regulated genes (*ERF1*), and lipoxygenase (*LOX*), and enhanced the accumulation of ROS, callose, and PAL [71]. StoVe1 is a *Ve1* homolog from *S. torvum*, and StoVe1-overexpressing potato lines show increased resistance to *V. dahliae* [72]. *StVe*, a potential Verticillium wilt disease resistance gene, from *S. torvum* and *SlVe1* from *S. lycopersicoides* are homologous to *Ve1* and *Ve2*, respectively, and *StVe* and *SlVe1* encode cell surface-like receptor proteins [73,74].

Micro-RNAs (miRNAs) are indispensable regulators of plant responses to biotic and abiotic stresses [75]. RNA silencing has an important role in plant defence against fungal pathogens and exerts specific defence functions against *V. dahliae* [76]. Upland cotton KV-1 displays multi-level resistance against *Verticillium* wilt, and some novel small RNAs have been identified after infection by *V. dahliae* strains with different virulence, V991 and D07038 [77]. Variants 3444a-5p and miR5562 showed the highest expression level in virulent conditions, whereas miR1423a-5p showed low-level expression [78]. Members of the potato miR482 superfamily and their variants were shown to target a class of disease-resistance proteins with nucleotide-binding sites and leucine-rich repeat (LRR) motifs, and miR482e-overexpressing transgenic potato plants show hypersensitivity to *V. dahliae* infection [79]. GhlncNAT-ANX2 and GhlncNAT-RLP7 are two species-conserved long noncoding RNAs, and their silencing in cotton significantly increases resistance to *V. dahliae*, which is possibly related to the upregulated expression of *lipoxygenase 1* and *lipoxygenase 2* [80]. Expression of *GbRLK*, a receptor-like kinase gene from the disease-resistant cotton *G. barbadense* Hai7124, is induced by *V. dahliae*. Moreover, transgenic cotton and the overexpression of GbRLK in *Arabidopsis* plants result in resistance against *V. dahliae* infection [17].

## 5. Transcription Factors

The MYB family of proteins is both large and diverse, with many members functioning as transcription factors. Increasing evidence shows that plant MYB transcription factors partake in defence against pathogen infection. Infection by *V. dahliae* stimulates Ca^2+^ influx into the cytosol and enhances GhMYB108 expression in cotton root cells. GhMYB108 was demonstrated as interacting with the calmodulin-like protein GhCML11 in a calcium-dependent manner; thus, *GhCML11* and *GhMYB108* silencing enhances cotton susceptibility to *V. dahliae* [81]. A stress-responsive HD-ZIP I transcription factor in cotton, GhHB12, negatively regulates the resistance of cotton plants by suppressing JA response genes [82]. CBP60g and SARD1 are two related plant-specific transcription factors involved in SA signalling. The secretory protein VdSCP41 in *V. dahliae* directly targets CBP60g to inhibit plant immunity, and mutations in *Arabidopsis CBP60g* and *SARD1* compromise resistance to *V. dahliae* [83]. A homeodomain transcription factor gene (HDTF1) was isolated from cotton, and silencing *HDTF1* was found to significantly enhance cotton plant resistance to *V. dahliae* and *Botrytis cinerea*, resulting in activation of JA-mediated signalling and JA accumulation [84]. Further, a basic helix-loop-helix (bHLH) transcription factor, GbbHLH171, interacts with and is phosphorylated by a defence-related receptor-like kinase in *G. barbadense*, namely, GbSOBIR1, in vitro and in vivo, and has a positive effect on cotton resistance to *V. dahliae* [85].

## 6. Signal Transduction

The ribosomal protein L18 (*GaRPL18*) gene from *G.arboreum* mediates plant resistance to *V. dahliae* via an SA-related signalling pathway. Silencing *GaRPL18* impairs cotton plants resistance to *V. dahliae*, whereas *GaRPL18* overexpression enhances *Arabidopsis* resistance to *V. dahliae* [86]. The phi-class glutathione (GSH) S-transferase gene (*GaGSTF9*) in *Gossypium arboreum* was found to be induced by *V. dahliae* via SA-related signalling pathway. *GaGSTF9* silencing in cotton enhances its susceptibility. Conversely, the overexpression of *GaGSTF9* increases *Arabidopsis* resistance to *V. dahliae* and the accumulation of endogenous SA and GST, indicating that GST might adjust ROS content via catalytic reduction of the tripeptide GSH, which in turn affects SA content [87].

Spermine (Spm) signalling is correlated with plant resistance to abiotic and biotic stresses. Two key rate-limiting enzymes in Spm biosynthesis are Spm synthase (GhSPMS) and S-adenosylmethionine decarboxylase (GhSAMDC), and *GhSAMDC* and *GhSPMS* silencing in cotton impairs plant resistance to *V. dahliae* infection. Enhanced resistance and the higher accumulation of Spm, SA, and leucine in transgenic *A. thaliana* overexpressing *GhSAMDC* suggest that *GhSAMDC* mediates Spm biosynthesis and contributes to plant resistance to *V. dahliae* via SA- and leucine-related signalling pathways [88]. The overexpression of cotton *GhACL5* in *Arabidopsis* increases both plant height and T-Spm levels. Moreover, *GhACL5* silencing in cotton results in a dwarf phenotype and also reduces resistance to *V. dahliae*. These results suggest that *GhACL5* expression is related to in planta levels of T-Spm and contributes to stem elongation and defence responses to *V. dahliae* [89]. A polyamine oxidase gene (PAO), which can catalyse the conversion of Spm to spermidine (Spd), is induced early and strongly via plant hormone application and inoculation with *V. dahliae*. The constitutive expression of *GhPAO* in *A. thaliana* enhances resistance against *V. dahliae* and results in the accumulation of high levels of hydrogen peroxide, SA, and camalexin (a phytoalexin). These results suggest that GhPAO contributes to plant resistance to *V. dahliae* by activating Spm and camalexin signalling pathways [90].

Cotton cyclin-dependent kinase E (*GhCDKE*) is induced in cotton via *V. dahliae* infection and MeJA treatment. GhCDKE increases plant resistance to *V. dahliae*, which is mediated by the JA response pathway. *GhCDKE* silencing in cotton enhances susceptibility to *Verticillium* wilt pathogen, whereas *GhCDKE* overexpression in *A. thaliana* increases resistance to the pathogen [91]. GbWRKY1, a negative regulator of the JA-mediated defence pathway, contributes to plant resistance against *B. cinerea* and *V. dahliae.* During plant infection by *V. dahliae*, GbWRKY1 is also a key regulator that mediates the plant defence-to-development transition by activating JAZ1 expression [92]. GhCPK33 from *G. hirsutum* negatively regulates cotton resistance to *V. dahliae* by directly controlling JA biosynthesis. *GhCPK33* silencing was shown to constitutively activate JA biosynthesis and JA mediated-defence responses and enhance resistance to *V. dahliae* infection [93]. GbSSI2 is an important regulator of the crosstalk between SA and JA signalling pathways. Moreover, the exogenous application of brassinolide can activate brassinosteroids and JA and enhance the resistance of cotton plants *to V. dahliae* [50].

Further, one study found that ET signalling in cotton roots infected with *V. dahliae* is significantly activated, which resulted in the high expression of ET biosynthesis and signal components [94]. The etr1-1 (ET receptor mutant) *Arabidopsis* strain shows enhanced resistance to *V. dahliae*, as well as enhanced activation and increased accumulation of CHI-1, CHI-2, GSTF12, GSTU16, Myb75, PR-1, PR-2, and PR-5 [95]. Ethylene-responsive factors (ERFs) commonly play an important role in pathogen defence responses. GbERFb, a new AP2/ERF transcription factor, can improve plant disease resistance [96]. GbERF1-like, ET response-related factor derived from *G. barbadense*, contributes to plant resistance against *V. dahliae* by positively regulating lignin synthesis. This resistance depends on the activation of *GhHCT1* and *AtPAL3* promoters by GbERF1-like proteins [97]. *GbABR1* is an ERF subfamily B4 member and a new member of the AP2 family from *G. barbadense*. *GbABR1*-silenced plants show a higher disease index, indicating that this gene positively regulates resistance to *Verticillium* wilt [98].

Nucleotide-binding site leucine-rich repeat (NBS-LRR) proteins play a key role in plant defence against pathogens. A genome-wide association study indicated that CG02-containing TIR-NBS-LRR domains are the most likely candidate underlying cotton resistance to *V. dahliae* [99]. The island cotton NBS-LRR gene *GbaNA1* can be induced by the pathogen *V. dahliae* and by the phytohormones ET, JA, and SA, contributing to island cotton resistance to *V. dahliae* isolate Vd991 [100]. The overexpression of *GbaNA1* in *A. thaliana* enhances ROS content and the expression of genes related to the ethylene signalling pathway [101].

Serine/threonine-protein kinase (STK) is involved in responses to pathogen infection and oxidative stress via phosphorylation. The overexpression of *G. barbadense GbSTK* in *Arabidopsis* enhances resistance to *V. dahliae* and elevates PR-4, PR-5, and EREBP expression [102]. Moreover, the mitogen-activated protein kinase (MAPK) cascade plays key roles in plant defence against pathogen attack. MKK members in MAPK signalling cascades play dual roles in subtly regulating the resistance of cotton plants to *Verticillium* wilt; *GhMKK4, GhMKK6*, and *GhMKK9* positively regulate, whereas GhMKK10 negatively regulates, cotton resistance [103]. As such, *GhMKK2* and *GhNDR1* silencing compromises the resistance of cotton plants to *V. dahliae* infection [104].

## 7. Concluding Remarks

Currently, numerous genes related to *V. dahliae* resistance have been identified in plants (Table 1). However, *Verticillium* wilt is still an enormous threat to agricultural production. Due to the presence of microsclerotia in *V. dahliae*, it can survive in the soil for many years, and it rapidly spreads; thus, it is difficult to control once infection occurs. Moreover, there is no fungicide available for plants, further limiting efficient control. Currently, the most effective control measures are genetic resistance. Therefore, it is necessary to further explore the genes related to *V. dahliae* resistance in plants. Resistance-related genes can be explored from various perspectives such as extracellular enzymes, the cell wall, PRRs, transcription factors, and SA/JA/ET-related signal transduction pathways. The further development of new technology such as host-induced gene silencing can aid in plant protection. At present, many genes remain unidentified, and there are few known genes related to resistance. Therefore, the technology available for genetic research is also extremely limited. In this paper, we summarised the genes related to *V. dahliae* resistance in plants on the basis of extracellular enzymes, cell walls, PRRs, transcription factors, and SA/JA/ET-related signal transduction pathways. This report provides a good theoretical resource for researchers and could aid in the discovery of additional genes related to resistance by providing a theoretical basis to further understand the molecular genetic mechanisms of plant resistance to *V. dahliae*. With the development of molecular biology technology and the application of multi-omics integrative analyses to the study plant disease resistance mechanisms, it is possible to study interactions between plants and Verticillium wilt, which will contribute to the discovery of plant disease resistance genes. At the same time, with the in-depth analysis of the molecular mechanisms underlying plant resistance to Verticillium wilt, it will be possible to obtain crops varieties that are resistant to Verticillium wilt through genetic engineering and breeding technology.

## Figures and Tables

**Table 1:** Genes related to plant resistance to *V. dahlia* and their regulatory mechanisms.

Classification	Protein (Gene) Name	Annotation	Host	Resistance Mechanism	References
**Defence-related proteins**	PGIP	plant defence protein	Ck, Gh	inhibit fungal polygalacturonase activity	[24]
	*NaD1*	plant defensin	Na	antifungal activity	[26]
	*GbNRX1*	apoplastic thioredoxin protein	Gb	apoplastic immune response and scavenge ROS	[27]
	*GbHyPRP1*	proline-rich protein	Gb	thickening cell walls and ROS accumulation	[28]
	*AtNPR1*	non-expressor of Pr1	At	upregulating expression of ICS1 and PR1a	[30]
	GhMLP28	defence-related major latex protein	St	enhance GhERF6 activity	[32]
	GbTLP1	thaumatin-like protein	Gb	secondary cell wall development	[34]
	BTD-S	synthetic defensin derivative	Synthetic	antifungal activity	[35,36]
	*StoCYP77A2*	cytochrome P450	Nt	synthesis of antimicrobial compounds	[37]
**Enzymes**	*Chi28*	class IV chitinase subfamily	Gh, Gb	degrade the fungal cell wall	[42]
	*GhPMEI3*	pectin methylesterases	Gh	degrade the fungal cell wall	[43]
	*GhLAC15*	laccase	Gh	lignification of the cell wall	[45]
	*GbSBT1*	a subtilase gene	Gb	activating defence-related genes expressionn	[46]
	*GhECR*	enoyl-CoA reductase	Gh	production of very long chain fatty acids	[47]
	*GbANS*	anthocyanidin synthase	Gb	regulating biosynthesis of anthocyanins	[48]
	*GhPUB17*	U-box E3 ubiquitin ligase	Gh	negatively regulating immunity	[49]
**Receptor-like proteins**	*GhDIR1*	putative dirigent protein	Gh	lignification of the cell wall	[38]
	*GhUMC1*	umecyanin-like protein	Gh		[39]
	*Lyp1, Lyk7* *,* *LysMe3*	lysin-motif receptor kinases	Gb	recognize chitin, receptor-mediated endocytosis-like signals and leucine zipper, enhance the expression of the JA/ET signalling pathway-related genes, increase the expressions of defence-related genes	[53]
	*Gh-LYK1* *,* *Gh-LYK2*		Gh		[54]
	*Ve1 and Ve2*	cell-surface glycoproteins	Sl		[16,25]
	*GbSOBIR1*	defence-related receptor-like kinases	Gb		[85]
	*Gbvdr3, Gbvdr6* *Gbve1, VvVe*, *StVe* *StoVe1,SlVe1, GbRLK*	Ve1 homologues	Gb, Vv St, Sl		[17,64,68,69,70,71,72,73,74]
	*miR482e*	miR482 superfamily	St	target disease-resistance proteins with NBS and LRR motifs	[79]
**Transcription factors**	*GhHB12*	HD-ZIP I transcription factor	Gh	suppressing JA-response genes	[82]
	*GhMYB108*	plant MYB transcription factors	Gh	enhance defence signalling molecules	[81]
	*CBP60g and SARD1*	plant-specific transcription factors	At	regulating SA signalling	[83]
**Signal transduction**	*GaRPL18*	ribosomal protein L18	Ga	mediate resistance by SA-signalling	[86]
	*GaGSTF9*	phi-class glutathione S-transferase	Ga	regulating ROS via catalytic reduction of glutathione	[87]
	*GhSAMDC* *,* *GhSPMS*	S-adenosylmethionine decarboxylase	Gh	regulating Spm biosynthesis by SA-signalling	[88]
	*GhPAO*	polyamine oxidase	Gh	regulating Spm and camalexin signalling	[90]
	*GhCDKE*	cyclin-dependent kinase	Gh	enhance plant resistance by JA pathway	[91]
	*HDTF1*	homeodomain transcription factor	Gh	activation of JA-mediated signalling	[84]
	*GbWRKY1*	regulator mediating	Gb	activating JAZ1 expression	[92]
	*GbSSI2,GbCAD1*	regulating signal pathways	Gb	activating JA-mediated signalling	[50]
	*GbaNA1*	NBS-LRR protein	Gb	regulating ROS and ET signalling pathway	[100,101]
	*ETR1*	ET receptor	At	activation and increased accumulation of defence proteins	[95]
	*GbERF1-like*	ET response-related factor	Gb	positive regulator in lignin synthesis	[97]

Notes: At, *Arabidopsis thaliana*; Ck, *Cynanchum komarovii*; ET, ethylene; Ga, *Gossypium arboreum*; Gb, *Gossypium barbadense*; Gh, *Gossypium hirsutum*; ICS, isochorismate synthase; JA, jasmonic acid; LncRNAs, long noncoding RNAs; Na, *Nicotiana alata*; Nt, *Nicotiana tabacum*; Pr, pathogenesis-related protein; ROS, reactive oxygen species; SA, salicylic acid; Spm, spermine; Sl, *Solanum lycopersicum*; St, *Solanum torvum*; T, threonine; Vv, *Vitis vinifera*.

## References

[1] Klosterman S.J., Atallah Z.K., Vallad G.E., Subbarao K.V. (2009). Diversity, pathogenicity; and management of *Verticillium* Species. Annu. Rev. Phytopathol..

[2] Fradin E.F., Thomma B.P. (2006). Physiology and molecular aspects of *Verticillium* wilt diseases caused by *V. dahliae* and *V. albo-atrum*. Mol. Plant Pathol..

[3] Wang X.K., Wang C.Y., Xie C.J., Yang X.Y. (2014). Advances in molecular mechanisms of *Verticillium* pathogenicity and plant resistance to *Verticillium* wilt. J. Henan. Agr. Sci..

[4] Zhang J., Fang H., Zhou H., Sanogo S., Ma Z. (2014). Genetics; breeding; and marker-assisted selection for Verticillium wilt resistance in cotton. Crop Sci..

[5] Prieto P., Navarroraya C., Valverdecorredor A., Amyotte S.G., Dobinson K.F., Mercadoblanco J., Segura A., Preston G., Wit P.D. (2010). Colonization process of olive tissues by *Verticillium dahliae* and its in planta interaction with the biocontrol root endophyte *Pseudomonas fluorescens* PICF7. Microb. Biotechnol..

[6] Zhao P., Zhao Y.L., Jin Y., Zhang T., Guo H.S. (2014). Colonization process of *Arabidopsis thaliana* roots by a green fluorescent protein-tagged isolate of *Verticillium dahliae*. Protein Cell.

[7] Shaban M., Miao Y., Ullah A., Khan A.H., Ahmed M.M., Tabassum M.A., Zhu L. (2018). Physiological and molecular mechanism of defense in cotton against *Verticillium dahliae*. Plant Physiol. Biochem..

[8] Temple S.H., DeVay J., Forrester L.L. (1973). Temperature effects upon development and pathogenicity of defoliating and nondefoliating pathotypes of *Verticillium dahliae* in leaves of cotton plants. Phytopathology.

[9] Daayf F. (2015). Verticillium wilts in crop plants: Pathogen invasion and host defence responses. Can. J. Plant Pathol..

[10] Talboys P.W. (1958). Association of tylosis and hyperplasia of the xylem with vascular invasion of the hop by *Verticillium albo-atrum*. Trans. Brit. Mycol. Soc..

[11] Meyer R., Slater V., Dubery I.A. (1994). A phytotoxic protein-lipopolysaccharide complex produced by *Verticillium dahliae*. Phytochemistry.

[12] Porter C., Green R. (1952). Production of exotoxin in the genus *Verticillium*. Phytopathology.

[13] Luo X., Xie C., Dong J., Yang X., Sui A. (2014). Interactions between *Verticillium dahliae* and its host: Vegetative growth, pathogenicity, plant immunity. Appl. Microbiol. Biotechnol..

[14] Belien T., Van C.S.J., Volckaert G. (2006). Microbial endoxylanases, effective weapons to breach the plant cell-wall barrier or, rather, triggers of plant defense systems?. Mol. Plant-Microbe Interact..

[15] Jones J.D., Dangl J.L. (2006). The plant immune system. Nature.

[16] Fradin E.F., Zhang Z., Rovenich H., Song Y., Liebrand T.W., Masini L., van den Berg G.C., Joosten M.H., Thomma B.P. (2014). Functional analysis of the tomato immune receptor Ve1 through domain swaps with its non-functional homolog Ve2. PLoS ONE.

[17] Zhao J., Zhang Z., Gao Y., Zhou L., Fang L., Chen X., Ning Z., Chen T., Guo W., Zhang T. (2015). Overexpression of GbRLK, a putative receptor-like kinase gene, improved cotton tolerance to *Verticillium* wilt. Sci. Rep..

[18] Bu B., Qiu D., Zeng H., Guo L., Yuan J., Yang X. (2014). A fungal protein elicitor PevD1 induces *Verticillium* wilt resistance in cotton. Plant Cell Rep..

[19] Liang Y., Cui S., Tang X., Zhang Y., Qiu D., Zeng H., Guo L., Yuan J., Yang X. (2018). An asparagine-rich protein Nbnrp1 modulate *Verticillium dahliae* protein PevD1-induced cell death and disease resistance in *Nicotiana benthamiana*. Front. Plant Sci..

[20] Liu M., Khan N.U., Wang N., Yang X., Qiu D. (2016). The protein elicitor PevD1 enhances resistance to pathogens and promotes growth in *Arabidopsis*. Int. J. Biol. Sci..

[21] Zhang Y., Gao Y., Liang Y., Dong Y., Yang X., Qiu D. (2019). *Verticillium dahliae* PevD1, an Alt a 1-like protein, targets cotton PR5-like protein and promotes fungal infection. J. Exp. Bot..

[22] Zhou R., Zhu T., Han L., Liu M., Xu M., Liu Y., Han D., Qiu D., Gong Q., Liu X. (2017). The asparagine-rich protein NRP interacts with the *Verticillium* effector PevD1 and regulates the subcellular localization of cryptochrome 2. J. Exp. Bot..

[23] Liu N., Ma X., Yun S., Zhang X., Li F., Hou Y. (2017). Necrotizing activity of *Verticillium dahliae* and *Fusarium oxysporum* f. sp. vasinfectum endopolygalacturonases in cotton. Plant Dis..

[24] Liu N., Zhang X., Sun Y., Wang P., Li X., Pei Y., Li F., Hou Y. (2017). Molecular evidence for the involvement of a polygalacturonase-inhibiting protein, GhPGIP1, in enhanced resistance to *Verticillium* and *Fusarium* wilts in cotton. Sci. Rep..

[25] Nazar R.N., Xu X., Kurosky A., Robb J. (2018). Antagonistic function of the *Ve* R-genes in tomato. Plant Mol. Biol..

[26] Gaspar Y.M., McKenna J.A., McGinness B.S., Hinch J., Poon S., Connelly A.A., Anderson M.A., Heath R.L. (2014). Field resistance to *Fusarium oxysporum* and *Verticillium dahliae* in transgenic cotton expressing the plant defensin NaD1. J. Exp. Bot..

[27] Li Y.B., Han L.B., Wang H.Y., Zhang J., Sun S.T., Feng D.Q., Yang C.L., Sun Y.D., Zhong N.Q., Xia G.X. (2016). The thioredoxin GbNRX1 plays a crucial role in homeostasis of apoplastic reactive oxygen species in response to *Verticillium dahliae* infection in cotton. Plant Physiol..

[28] Yang J., Zhang Y., Wang X., Wang W., Li Z., Wu J., Wang G., Wu L., Zhang G., Ma Z. (2018). HyPRP1 performs a role in negatively regulating cotton resistance to *Verticillium dahliae* via the thickening of cell walls and ROS accumulation. BMC Plant Boil..

[29] Pajerowska-Mukhtar K.M., Emerine D.K., Mukhtar M.S. (2013). Tell me more: Roles of NPRs in plant immunity. Trends Plant Sci..

[30] Parkhi V., Kumar V., Campbell L.A.M., Bell A.A., Rathore K.S. (2010). Expression of *Arabidopsis* NPR1 in transgenic cotton confers resistance to non-defoliating isolates of *Verticillium dahliae* but not the defoliating isolates. J. Phytopathol..

[31] Jue D., Liu Y., Shi C., Min C., Yang Q. (2014). Cloning and characterization of a *Solanum torvum* NPR1 gene involved in regulating plant resistance to *Verticillium dahliae*. Acta Physiol. Plant..

[32] Yang C.L., Liang S., Wang H.Y., Han L.B., Wang F.X., Cheng H.Q., Wu X.M., Qu Z.L., Wu J.H., Xia G.X. (2015). Cotton major latex protein 28 functions as a positive regulator of the ethylene responsive factor 6 in defense against *Verticillium dahliae*. Mol. Plant..

[33] Fu X., Li C., Zhou X., Liu S., Wu F. (2016). Physiological response and sulfur metabolism of the *V. dahliae*-infected tomato plants in tomato/potato onion companion cropping. Sci. Rep..

[34] Munis M.F., Tu L., Deng F., Tan J., Xu L., Xu S., Long L., Zhang X. (2010). A thaumatin-like protein gene involved in cotton fiber secondary cell wall development enhances resistance against *Verticillium dahliae* and other stresses in transgenic tobacco. Biochem. Biophys. Res. Commun..

[35] Li F., Shen H., Wang M., Fan K., Bibi N., Ni M., Yuan S., Wang X. (2016). A synthetic antimicrobial peptide BTD-S expressed in *Arabidopsis thaliana* confers enhanced resistance to *Verticillium dahliae*. Mol. Genet. Genom..

[36] Ni M., Zhao Y., Bibi N., Shao M., Yuan S., Fan K., Zhang G., Li F., Wang X. (2013). A non-cyclic baboon θ-defensin derivative exhibiting antimicrobial activity against the phytopathogen *Verticillium dahliae*. Appl. Microbiol. Bio..

[37] Liu Y., Shi C., Mu X., Chao L., Ke S., Zhu W., Yang Q. (2015). Cloning and expression of a wild eggplant cytochrome P450 gene, *StoCYP77A2*, involved in plant resistance to *Verticillium dahliae*. Plant Biotech. Rep..

[38] Shi H., Liu Z., Zhu L., Zhang C., Chen Y., Zhou Y., Li F., Li X. (2012). Overexpression of cotton (*Gossypium hirsutum*) *dirigent1* gene enhances lignification that blocks the spread of *Verticillium dahliae*. Acta Biochim. Biophys. Sin..

[39] Zhu W., Gao E., Shaban M., Wang Y., Wang H., Nie X., Zhu L. (2018). GhUMC1, a blue copper-binding protein; regulates lignin synthesis and cotton immune response. Biochem. Bioph. Res. Commun..

[40] Xu J., Xu X., Tian L., Wang G., Zhang X., Wang X., Guo W. (2016). Discovery and identification of candidate genes from the chitinase gene family for *Verticillium dahliae* resistance in cotton. Sci. Rep..

[41] Cheng X.X., Zhao L.H., Klosterman S.J., Feng H.J., Feng Z.L., Feng W., Shi Y.Q., Li Z.F., Zhu H.Q. (2017). The endochitinase VDECH from *Verticillium dahliae* inhibits spore germination and activates plant defense responses. Plant Sci..

[42] Han L.B., Li Y.B., Wang F.X., Wang W.Y., Liu J., Wu J.H., Zhong N.Q., Wu S.J., Jiao G.L., Wang H.Y. (2019). The cotton apoplastic protein CRR1 stabilizes chitinase 28 to facilitate defense against the fungal pathogen *Verticillium dahliae*. Plant Cell.

[43] Liu N., Sun Y., Pei Y., Zhang X., Wang P., Li X., Li F., Hou Y. (2018). A pectin methylesterase inhibitor enhances resistance to *Verticillium* wilt. Plant Physiol..

[44] Xu L., Zhu L., Tu L., Liu L., Yuan D., Jin L., Long L., Zhang X. (2011). Lignin metabolism has a central role in the resistance of cotton to the wilt fungus *Verticillium dahliae* as revealed by RNA-Seq-dependent transcriptional analysis and histochemistry. J. Exp. Bot..

[45] Zhang Y., Wu L., Chen B., Zhao J., Cui J., Li Z., Yang J., Wu L., Zhang G., Ma Z. (2018). The cotton laccase gene *GHLAC15* enhances *Verticillium* wilt via an increase in defence-induced lignification and lignin components in the cell walls of plants. Mol. Plant Pathol..

[46] Duan X., Zhang Z., Wang J., Zuo K. (2016). Characterization of a novel cotton subtilase gene *GbSBT1* in response to extracellular stimulations and its role in *Verticillium* resistance. PLoS ONE.

[47] Mustafa R., Hamza M., Kamal H., Mansoor S., Scheffler J., Amin I. (2017). tobacco rattle virus-based silencing of enoyl-CoA reductase gene and its role in resistance against cotton wilt disease. Mol. Biotechnol..

[48] Long L., Zhao J.R., Xu F.C., Yang W.W., Liao P., Gao Y., Gao W., Song C.P. (2018). Silencing of *GbANS* reduces cotton resistance to *Verticillium dahliae* through decreased ROS scavenging during the pathogen invasion process. Plant Cell Tiss. Org..

[49] Qin T., Liu S., Zhang Z., Sun L., He X., Lindsey K., Zhu L., Zhang X. (2019). GhCyP3 improves the resistance of cotton to *Verticillium dahliae* by inhibiting the E3 ubiquitin ligase activity of GhPUB17. Plant Mol. Biol..

[50] Gao W., Long L., Zhu L.F., Xu L., Gao W.H., Sun L.Q., Liu L.L., Zhang X.L. (2013). Proteomic and virus-induced gene silencing (VIGS) analyses reveal that gossypol, brassinosteroids, and jasmonic acid contribute to the resistance of cotton to *Verticillium dahliae*. Mol. Cell Proteomics.

[51] Tanaka K., Nguyen C.T., Liang Y., Cao Y., Stacey G. (2013). Role of LysM receptors in chitin-triggered plant innate immunity. Plant Signal Behav..

[52] Wan J., Tanaka K., Zhang X.C., Son G.H., Brechenmacher L., Nguyen T.H., Stacey G. (2012). LYK4, a lysin motif receptor-like kinase, is important for chitin signaling and plant innate immunity in *Arabidopsis*. Plant Physiol..

[53] Xu J., Wang G., Wang J., Li Y., Tian L., Wang X., Guo W. (2017). The lysin motif-containing proteins, Lyp1, Lyk7 and LysMe3, play important roles in chitin perception and defense against *Verticillium dahliae* in cotton. BMC Plant Biol..

[54] Gu Z., Liu T., Ding B., Li F., Wang Q., Qian S., Ye F., Chen T., Yang Y., Wang J. (2017). Two Lysin-motif receptor kinases; Gh-LYK1 and Gh-LYK2; contribute to resistance against *Verticillium* wilt in upland cotton. Front. Plant Sci..

[55] Nazar R.N., Castroverde C.D.M., Xu X., Kurosky A., Robb J. (2019). Wounding induces tomato *Ve1* R-gene expression. Planta.

[56] Kawchuk L.M., Hachey J., Lynch D.R., Kulcsar F., van Rooijen G., Waterer D.R., Robertson A., Kokko E., Byers R., Howard R.J. (2001). Tomato *Ve* disease resistance genes encode cell surface-like receptors. Proc. Natl. Acad. Sci. USA.

[57] Castroverde C.D., Xu X., Blaya F.J., Nazar R.N., Robb J. (2017). Epistatic influence in tomato *Ve1*-mediated resistance. Plant Biol..

[58] De Jonge R., van Esse H.P., Maruthachalam K., Bolton M.D., Santhanam P., Saber M.K., Zhang Z., Usami T., Lievens B., Subbarao K.V. (2012). Tomato immune receptor Ve1 recognizes effector of multiple fungal pathogens uncovered by genome and RNA sequencing. Proc. Natl. Acad. Sci. USA.

[59] Castroverde C.D., Xu X., Nazar R.N., Robb J. (2017). Biotic factors that induce the tomato Ve1 R-gene. Plant Sci..

[60] Gayoso C., Pomar F., Novo-Uzal E., Merino F., de Ilarduya O.M. (2010). The Ve-mediated resistance response of the tomato to *Verticillium dahliae* involves H_2_O_2_, peroxidase and lignins and drives PAL gene expression. BMC Plant Biol..

[61] Fradin E.F., Abd-El-Haliem A., Masini L., van den Berg G.C., Joosten M.H., Thomma B.P. (2011). Interfamily transfer of tomato *Ve1* mediates *Verticillium* resistance in *Arabidopsis*. Plant Physiol..

[62] Fradin E.F., Zhang Z., Juarez-Ayala J.C., Castroverde C.D., Nazar R.N., Robb J., Liu C.M., Thomma B.P. (2009). Genetic dissection of *Verticillium* wilt resistance mediated by tomato Ve1. Plant Physiol..

[63] Song Y., Liu L., Wang Y., Valkenburg D.J., Zhang X., Zhu L., Thomma B. (2018). Transfer of tomato immune receptor Ve1 confers Ave1-dependent *Verticillium* resistance in tobacco and cotton. Plant Biotechnol. J..

[64] Zhang Z., Fradin E., de Jonge R., van Esse H.P., Smit P., Liu C.M., Thomma B.P. (2013). Optimized agroinfiltration and virus-induced gene silencing to study Ve1-mediated *Verticillium* resistance in tobacco. Mol. Plant Microbe. Interact..

[65] Zhang Z., van Esse H.P., van Damme M., Fradin E.F., Liu C.M., Thomma B.P. (2013). Ve1-mediated resistance against *Verticillium* does not involve a hypersensitive response in *Arabidopsis*. Mol. Plant Pathol..

[66] Song Y., Zhang Z., Seidl M.F., Majer A., Jakse J., Javornik B., Thomma B.P. (2017). Broad taxonomic characterization of *Verticillium* wilt resistance genes reveals an ancient origin of the tomato Ve1 immune receptor. Mol. Plant Pathol..

[67] Zhang B., Yang Y., Chen T., Yu W., Liu T., Li H., Fan X., Ren Y., Shen D., Liu L. (2012). Island cotton *Gbve1* gene encoding a receptor-like protein confers resistance to both defoliating and non-defoliating isolates of *Verticillium dahliae*. PLoS ONE.

[68] Yang Y., Chen T., Ling X., Ma Z. (2017). *Gbvdr6*, a gene encoding a receptor-like protein of cotton (*Gossypium barbadense*), confers resistance to *Verticillium* wilt in *Arabidopsis* and upland cotton. Front. Plant Sci..

[69] Zhang B.J., Zhang H.P., Chen Q.Z., Tang N., Wang L.K., Wang R.F., Zhang B.L. (2016). Molecular cloning and analysis of a receptor-like promoter of *Gbvdr3* gene in sea island cotton. Genet. Mol. Res..

[70] Chen T., Kan J., Yang Y., Ling X., Chang Y., Zhang B. (2006). A Ve homologous gene from *Gossypium barbadense*, *Gbvdr3*, enhances the defense response against *Verticillium dahliae*. Plant Physiol. Biochem..

[71] Tang J., Lin J., Yang Y., Chen T., Ling X., Zhang B., Chang Y. (2016). Ectopic expression of a Ve homolog *VvVe* gene from *Vitis vinifera* enhances defense response to *Verticillium dahliae* infection in tobacco. Gene.

[72] Liu S., Zhu Y., Xie C., Jue D., Hong Y., Chen M., Hubdar A.K., Yang Q. (2012). Transgenic potato plants expressing *StoVe1* exhibit enhanced resistance to *Verticillium dahliae*. Plant Mol. Biol. Rep..

[73] Chai Y., Zhao L., Liao Z., Sun X., Zuo K., Zhang L., Wang S., Tang K. (2003). Molecular cloning of a potential *Verticillium dahliae* resistance gene *SlVe1* with multi-site polyadenylation from *Solanum licopersicoides*. DNA Seq..

[74] Fei J., Chai Y., Wang J., Lin J., Sun X., Sun C., Zuo K., Tang K. (2004). cDNA cloning and characterization of the Ve homologue gene StVe from *Solanum torvum* Swartz. DNA Seq..

[75] Jones-Rhoades M.W., Bartel D.P. (2004). Computational identification of plant microRNAs and their targets; including a stress-induced miRNA. Mol. Cell.

[76] Ellendorff U., Fradin E.F., Thomma B.P., De Jonge R. (2008). RNA silencing is required for *Arabidopsis* defence against *Verticillium* wilt disease. J. Exp. Bot..

[77] He X., Sun Q., Jiang H., Zhu X., Mo J., Long L., Xiang L., Xie Y., Shi Y., Yuan Y. (2014). Identification of novel microRNAs in the *Verticillium* wilt-resistant upland cotton variety KV-1 by high-throughput sequencing. SpringerPlus.

[78] He X.H., Shi M., Sun Q., Cai Y.F. (2014). Expression profile analysis and target gene prediction of three conserved MicroRNAs in resistant Gossypium hirsutum cv. KV-1 responding to Verticillium dahlia. Advanced Materials Research.

[79] Yang L., Mu X., Liu C., Cai J., Shi K., Zhu W., Yang Q. (2015). Overexpression of potato *miR482e* enhanced plant sensitivity to *Verticillium dahliae* infection. J. Integ. Plant. Boil..

[80] Zhang L., Wang M., Li N., Wang H., Qiu P., Pei L., Xu Z., Wang T., Gao E., Liu J. (2018). Long noncoding RNA s involve in resistance to *Verticillium dahliae*; a fungal disease in cotton. Plant Biotechnol. J..

[81] Cheng H.Q., Han L.B., Yang C.L., Wu X.M., Zhong N.Q., Wu J.H., Wang F.X., Wang H.Y., Xia G.X. (2016). The cotton MYB108 forms a positive feedback regulation loop with CML11 and participates in the defense response against *Verticillium dahliae* infection. J. Exp. Bot..

[82] He X., Wang T., Zhu W., Wang Y., Zhu L. (2018). GhHB12, a HD-ZIP I transcription factor, negatively regulates the cotton resistance to *Verticillium dahliae*. Int. J. Mol. Sci..

[83] Qin J., Wang K., Sun L., Xing H., Wang S., Li L., Chen S., Guo H.S., Zhang J. (2018). The plant-specific transcription factors CBP60g and SARD1 are targeted by a *Verticillium* secretory protein VdSCP41 to modulate immunity. eLife.

[84] Gao W., Long L., Xu L., Lindsey K., Zhang X., Zhu L. (2016). Suppression of the homeobox gene HDTF1 enhances resistance to *Verticillium dahliae* and *Botrytis cinerea* in cotton. J. Integr. Plant Biol..

[85] Zhou Y., Sun L., Wassan G.M., He X., Shaban M., Zhang L., Zhu L., Zhang X. (2019). GbSOBIR1 confers *Verticillium* wilt resistance by phosphorylating the transcriptional factor GbbHLH171 in *Gossypium barbadense*. Plant Biotechnol. J..

[86] Gong Q., Yang Z., Wang X., Butt H.I., Chen E., He S., Zhang C., Zhang X., Li F. (2017). Salicylic acid-related cotton (*Gossypium arboreum*) ribosomal protein GaRPL18 contributes to resistance to *Verticillium dahliae*. BMC Plant Biol..

[87] Gong Q., Yang Z., Chen E., Sun G., He S., Butt H.I., Zhang C., Zhang X., Yang Z., Du X. (2018). A Phi-class glutathione S-transferase gene for *Verticillium* wilt resistance in *Gossypium arboreum* identified in a genome-wide association study. Plant Cell Physiol..

[88] Mo H.J., Sun Y.X., Zhu X.L., Wang X.F., Zhang Y., Yang J., Yan G.J., Ma Z.Y. (2016). Cotton S-adenosylmethionine decarboxylase-mediated spermine biosynthesis is required for salicylic acid- and leucine-correlated signaling in the defense response to *Verticillium dahliae*. Planta.

[89] Mo H., Wang X., Zhang Y., Yang J., Ma Z. (2015). Cotton ACAULIS5 is involved in stem elongation and the plant defense response to *Verticillium dahliae* through thermospermine alteration. Plant Cell Rep..

[90] Mo H., Wang X., Zhang Y., Zhang G., Zhang J., Ma Z. (2015). Cotton polyamine oxidase is required for spermine and camalexin signalling in the defence response to *Verticillium dahliae*. Plant J..

[91] Li X., Pei Y., Sun Y., Liu N., Wang P., Liu D., Ge X., Li F., Hou Y. (2018). A cotton cyclin-dependent kinase E confers resistance to *Verticillium dahliae* mediated by jasmonate-responsive pathway. Front. Plant Sci..

[92] Li C., He X., Luo X., Xu L., Liu L., Min L., Jin L., Zhu L., Zhang X. (2014). Cotton WRKY1 mediates the plant defense-to-development transition during infection of cotton by *Verticillium dahliae* by activating JASMONATE ZIM-DOMAIN1 expression. Plant Physiol..

[93] Hu Q., Zhu L., Zhang X., Guan Q., Xiao S., Min L., Zhang X. (2018). *GhCPK33* negatively regulates defense against *Verticillium dahliae* by phosphorylating GhOPR3. Plant Physiol..

[94] Wang F.X., Ma Y.P., Yang C.L., Zhao P.M., Yao Y., Jian G.L., Luo Y.M., Xia G.X. (2011). Proteomic analysis of the sea-island cotton roots infected by wilt pathogen *Verticillium dahliae*. Proteomics.

[95] Pantelides I.S., Tjamos S.E., Paplomatas E.J. (2010). Ethylene perception via ETR1 is required in *Arabidopsis* infection by *Verticillium dahliae*. Mol. Plant Pathol..

[96] Liu J., Wang Y., Zhao G., Zhao J., Du H., He X., Zhang H. (2017). A novel *Gossypium barbadense* ERF transcription factor, GbERFb, regulation host response and resistance to *Verticillium dahliae* in tobacco. Physiol. Mol. Biol. Plant..

[97] Guo W., Jin L., Miao Y., He X., Hu Q., Guo K., Zhu L., Zhang X. (2016). An ethylene response-related factor; GbERF1-like; from *Gossypium barbadense* improves resistance to *Verticillium dahliae* via activating lignin synthesis. Plant Mol. Biol..

[98] Liu Y., Xin L., Lu L., Wang W., Quan S., Bo L., Wang C., Cheng J., Zhang Y., Xie Y. (2018). GbABR1 is associated with *Verticillium* wilt resistance in cotton. Biologia..

[99] Li T., Ma X., Li N., Zhou L., Liu Z., Han H., Gui Y., Bao Y., Chen J., Dai X. (2017). Genome-wide association study discovered candidate genes of *Verticillium* wilt resistance in upland cotton (*Gossypium hirsutum* L). Plant Biotechnol. J..

[100] Li N.Y., Ma X.F., Short D.P.G., Li T.G., Zhou L., Gui Y.J., Kong Z.Q., Zhang D.D., Zhang W.Q., Li J.J. (2018). The island cotton NBS-LRR gene *GbaNA1* confers resistance to the non-race 1 *Verticillium dahliae* isolate Vd991. Mol. Plant Pathol..

[101] Li N.Y., Zhou L., Zhang D.D., Klosterman S.J., Li T.G., Gui Y.J., Kong Z.Q., Ma X.F., Short D.P.G., Zhang W.Q. (2018). Heterologous expression of the cotton NBS-LRR gene *GbaNA1* enhances *Verticillium* wilt resistance in *Arabidopsis*. Front. Plant Sci..

[102] Zhang Y., Wang X., Li Y., Wu L., Zhou H., Zhang G., Ma Z. (2013). Ectopic expression of a novel Ser/Thr protein kinase from cotton (*Gossypium barbadense*), enhances resistance to *Verticillium dahliae* infection and oxidative stress in *Arabidopsis*. Plant Cell Rep..

[103] Meng J., Gao H., Zhai W., Shi J., Zhang M., Zhang W., Jian G., Zhang M., Qi F. (2018). Subtle regulation of cotton resistance to *Verticillium* wilt mediated by MAPKK family members. Plant Sci..

[104] Gao X., Wheeler T., Li Z., Kenerley C.M., He P., Shan L. (2011). Silencing GhNDR1 and GhMKK2 compromises cotton resistance to *Verticillium* wilt. Plant J..

